# Stereotactic body radiation therapy induces fast tumor control and symptom relief in patients with iliac lymph node metastasis

**DOI:** 10.1038/srep37987

**Published:** 2016-11-29

**Authors:** Zhongqiu Wang, Jing Wang, Hongqing Zhuang, Ping Wang, Zhiyong Yuan

**Affiliations:** 1 Department of Radiation Oncology and CyberKnife Center, Key Laboratory of Cancer Prevention and Therapy, Tianjin’s Clinical Research Center for Cancer, Tianjin Medical University Cancer Institute and Hospital, National Clinical Research Center for Cancer, Tianjin 300060, China

## Abstract

The CyberKnife is a robotic stereotactic body radiotherapy (SBRT) system which has shown promising results for many malignances with good efficacy and low toxicity. This study aims to evaluate the response and local control (LC) obtained with CyberKnife in the management of iliac lymph node metastases (ILNM). Twenty-two patients with 27 ILNM were treated by CyberKnife from May 2010 to May 2016. Median follow-up time was 33 months (8–97). The complete response, partial response, stable disease and progression disease rates were 37.0%, 48.0%, 7.5% and 7.5% respectively. The 1-, 2-, and 3-year LC rates were all 90.6%, and overall survival rates were 78.8%, 60.6%, and 43.3% respectively. All patients with pelvic pain and ureter obstruction achieved good and fast symptom relief, while leg edema persisted in 2 patients. The general treatment tolerance was acceptable and no severe toxicities were reported. No factors were found correlated with local failure. While overall survival (OS) was better for patients who had received a total dose more than 30 Gy or prior systemic treatment, and whose symptoms were relieved. Taken together, CyberKnife is an effective therapeutic option for ILNM, providing high LC rate and good symptom relief with minimal toxicity.

The iliac lymph nodes are one of the most frequently involved nodes by metastases, such as cervical cancer, rectal cancer, and ovarian cancer. Patients with iliac nodal metastases (ILMN) always present with evident symptoms such as leg swollen, back/ pelvic pain and ureter obstruction/ stenosis, which led to a greatly compromised quality of life and often a low survival rate^1^,^2^,^3^. It is widely known that salvage chemotherapy and surgery were the only two methods that have been commonly used to treat ILNM in the clinical settings. However, each method has its only limitations^4^. Regimens for salvage chemotherapy have to depend on histopathology of the primary tumor, and the results are disappointing. Surgery is considered as a curative option for some isolated metastases, but the lymphadenectomy sometimes can be difficult because of disease extent, involvement of critical structures and perioperative complications^4^. Therefore, there is a critical requirement to find other available treatments that are effective, safe and non-invasive for these lesions.

Recently, growing evidences have supported that stereotactic body radiotherapy (SBRT) is a safe, non-invasive, and effective technology, showing high local control (LC) rate and low incidence of complication in selected metastatic patients, especially when surgery is not allowed or refused^5^,^6^,^7^. It is defined by the American Society of Radiation Oncology as external-beam radiotherapy used to precisely deliver high doses of radiation to an extracranial target within the body, either as a single dose or a small limited number of radiation fractions^8^. The dose can be delivered either using a traditional linear accelerator or using a robotic arm (i.e. CyberKnife^®^). The CyberKnife was developed back in the 1990 s at Stanford (Accuray Incorporated, Sunnyvale, CA, USA)^9^, and represented a major paradigm shift from traditional stereotactic radiosurgery technology. It is capable of detecting and correcting for intrafraction tumor motion, as well as adapting to the patient’s breathing pattern and moving the linear accelerator in concert^10^. Therefore it can deliver dose at any point within the target and disperse the dose among normal tissues.

The therapeutic indications of CyberKnife previously included a variety of different tumors, such as lung cancer^11^, liver cancer^7^, pancreatic cancer^12^, and colorectal cancer^13^. However, very little is known about its clinical use for the treatment of ILNM. The aim of the present study was to assess the efficacy and safety of SBRT using the CyberKnife system for patients with ILNM.

## Results

### Patient and treatment characteristics

A total of 22 patients (27 treatment) with a median age of 53 years (range 37 to 87), including 5 males and 17 females, were treated using CyberKnife. Clinical data were collected. Primary tumor sites included cervical cancer (10 cases/45.5%), rectal cancer (4 cases/18.2%), ovarian cancer (2 cases/9.1%), ureter carcinoma (2 cases/9.1%), hodgkin lymphoma (2 cases/9.1%), duodenal cancer (1 case/4.5%), and renal pelvic carcinoma (1 case/4.5%). Among all the patients, nine (40.9%) presented with site metastases other than iliac lymph node. Fourteen (63.6%) and 19 (86.4%) had received external beam radiation therapy or chemotherapy before CyberKnife respectively, and three patients (13.6%) had more than one lesions treated. Besides, 7 patients (31.8%) were symptom-free, and 15 (68.2%) had symptoms such as back/ pelvic pain, leg edema and ureter obstruction. Demographic characteristics of patients were listed in Table 1.

**Figure 1:**
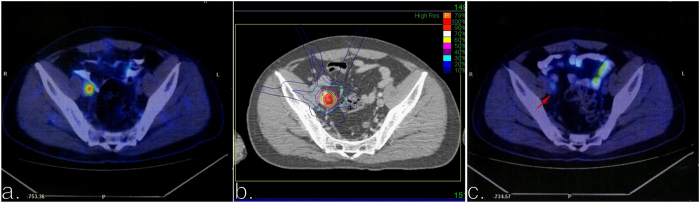
Examples of dose distributions and treatment outcome for one patient. (**a**) PET-CT scan before CyberKnife. (**b**) Treatment plan with 45 Gy in five fractions. (**c**) PET-CT scan three months after CyberKnife.

The median total dose was 39 Gy (range 21–51 Gy), which was delivered in 5 fractions (3–8 fractions), and corresponded to a biologic equivalent dose (BED_10_) of 72.6 Gy (35.7–100 Gy). The median PTV was 18.87 ml (0.88–125.66 ml), and the median prescription isodose line was 72% (64–82%). Summary of the treatment planning parameters was shown in Table 2. Figure 1 shows the representative PET-CT scan before (a) and three-month after (c) CyberKnife, and the corresponding treatment plan (b) for one of the patients treated with 45 Gy in five fractions (BED_10_ = 85.5 Gy).

### Treatment efficacy

**Figure 2:**
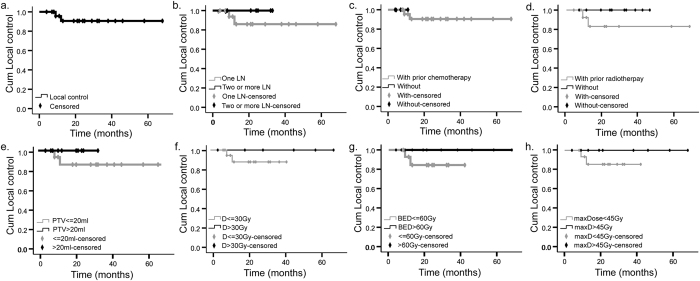
Actuarial local control of patients. (**a**) Overall local control. (**b**) Local control depending on involved lymph nodes (LN) per patient. (**c**) Local control depending on prior chemotherapy. (**d**) Local control depending on prior radiotherapy. (**e**) Local control depending on planning target volume (PTV). (**f**) Local control depending on prescribed dose. (**g**) Local control depending on biologic equivalent dose (BED_10_). (**h**) Local control depending on max dose to tumor. Cum, cumulative; D, dose.

**Figure 3:**
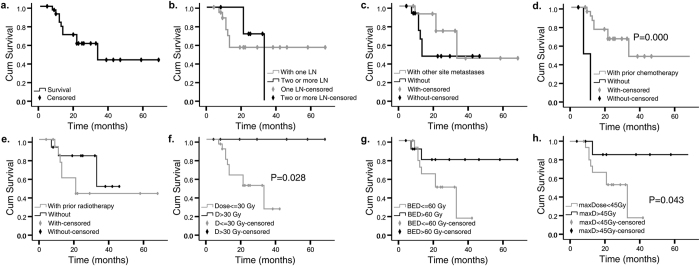
Actuarial overall survival of patients. (**a**) Overall survival in general. (**b**) Overall survival depending on involved lymph nodes (LN) per patient. (**c**) Overall survival depending on concurrence of other site metastasis. (**d**) Overall survival depending on prior chemotherapy. (**e**) Local control depending on prior radiotherapy. (**f**) Overall survival depending on prescribed dose. (**g**) Overall survival depending on biologic equivalent dose (BED_10_). (**h**) Overall survival depending on max dose to tumor. Cum, cumulative; D, dose; yr, year.

All patients were followed up until death or May 2016. By the last follow-up, eight patients (36.4%) died, leaving 14 patients in the final analysis. The median follow-up was 33 months (8–97 months), and median OS was 21 months (4–68 months). The 1-, 2-, and 3-year actuarial LC rates were all 90.6% (Fig. 2a). And 1-, 2-, and 3-year OS rates were 78.8%, 60.6% and 43.4% respectively (Fig. 3a). Using the RECIST standard, complete response, partial response and stable disease rates were 37%, 48%, and 7.5% respectively, while 7.5% patients had disease progression.

**Figure 4:**
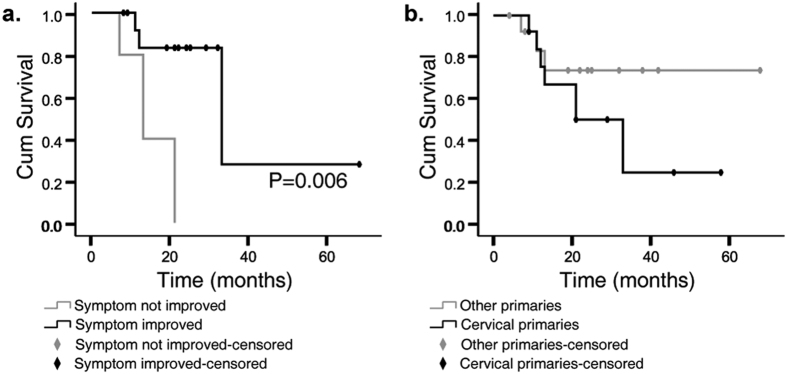
Overall survival of patients depending on symptom relief (**a**) and histological primaries (**b**).

In the univariate and multivariate analysis, there was no significant association between any of the factors and LC rates (Fig. 2b–h). However, patients who had undergone chemotherapy before CyberKnife had a better outcome than those who had not (Fig. 3d, P = 0.000). Furthermore, those underwent a total dose that are greater than 30 Gy had a longer OS compared with those were not (Fig. 3f, P = 0.028). Survival was also better for patients receiving a BED >60 Gy than those receiving a lower BED, although the difference was not statistically significant (Fig. 3g, P = 0.073). Since prescription isodose line varied greatly, we further analyzed max dose to tumor. Higher max dose, greater than 45 Gy, were associated with better OS (Fig. 3h, P = 0.043). Among the fifteen patients who had associated symptoms prior to the treatment (including pain (n = 12), edema (n = 2), or ureter obstruction (n = 1)), 13 of them (86.7%) had alleviated symptom after CyberKnife treatment. Pain was alleviated rapidly. More than a half of the patients achieved pain relief in the end of the treatment (n = 7), and the rest patients reported improvement at one-month follow-up (n = 5). One patient had both leg pain and edema. His pain disappeared at the second fraction of the radiation, and swelling disappeared at two weeks post-radiation. For the other two patients presented with led edema prior to the treatment, the symptom both reduced within one month of follow-up. When the associated symptoms improved, the OS was significantly higher (Fig. 4a, P = 0.006).

Moreover, when further compared between different types of cancers, LC and OS rates were not significantly different. For example, 1-year LC and OS rates were 83.1% and 75.5% for cervical primaries, while for non-cervical primaries, they were 100% and 83.1% respectively (Fig. 4b, P = 0.179).

### Treatment toxicity

The treatment was well-tolerated by all the patients. No grade 3 or higher toxicity was observed. Nausea, vomiting, fatigue and pain at the time of treatment were the most common side effects. The side effects were transitory and didn’t prevent any patient from completing the treatment. Summary of toxicities are listed in Table 3.

## Discussion

Our study described the application of CyberKnife, a form of SBRT, in treating patients with ILNM. Overall, CyberKnife is able to provide high rates of local tumor control and symptom relief without significant toxicity. The median survival period was 21 months, and the 1-, 2-, 3-year survival rates were 78.8%, 60.6%, and 43.3% respectively. LC rate was 90.6%, and symptom relief rate was 86.7%. Otherwise, no grade 3 or above acute toxicities were reported. Thus, it confirmed a promising role of CyberKnife in the clinical use for these patients, especially for a palliative purpose to resolve various symptoms caused by the metastases.

Recently, SBRT including CyberKnife has been applied as primary management option for patients with many metastatic diseases, such as lung, liver, adrenal, brain and bone metastases. Piet Ost *et al*.^14^ reported a series of 119 patients with oligometastatic prostate cancer in a multi-institutional analysis. The median progression-free survival was 21 months; a lower radiotherapy dose (≤100 Gy) predicted a higher local recurrence rate, and no grade >3 toxicity occurred. Ingrid Fumagalli^15^ reported the results of 113 liver and 26 lung metastases treated with SBRT: LC rates at 1- and 2-year were 84.5% and 66.1%, and the corresponding disease-free survival rates were 27% and 10% respectively; OS is comparable with other available options; and treatment is well tolerated. In our previous study, we recruited 57 patients with liver metastases. After treating using CyberKnife, the 1- and 2-year LC rates were 94.4% and 89.7% respectively. The median OS was 37.5 months for the whole group. In groups with primary tumors originating from colon, breast, stomach and sarcomas, the 2-year OS rate was 72.2%; and in groups with tumors from pancreas, lung, ovary, gallbladder, uterus, hepatocellular carcinoma, and olfactory neuroblastoma, the 2-year OS rate was 55.9%^16^. The findings from current study are consistent with previous studies. Moreover, it is the first to report the safety and efficacy of CyberKnife for a new setting of inoperable iliac lymph node oligometastases.

Systemic therapies are effective and critical for progressive disease, but a single treatment is often unlikely and unsatisfying to provide the long-term cure. Our results showed that patients who had undergone prior systemic treatment had a better LC and longer OS than those who had not, consistent with previous studies on liver or breast metastases^16^,^17^,^18^. Despite different types of medications, cycles of chemotherapy and the time interval between chemotherapy and CyberKnife, whether a combined systemic treatment before CyberKnife is placed seems to contribute to a favorable outcome of patients. Further investigation is needed.

Moreover, a total dose more than 30 Gy might also improve the patients’ survival. And those who received a BED more than 60 Gy had a longer OS compared with those whose BED was lower. Thus for a five-fraction regimen of SBRT, a prescription dose of at least 35 Gy should be considered.

The primary tumor sites of ILNM vary, which might affect the effectiveness of the usage of CyberKnife. Takeda A *et al*.^19^ reported that tumor origin was prognostically significant for metastatic lung tumors. The LC rate in colorectal-originated metastatic was significantly worse than that in lung metastases from other origins. However, our results indicated that LC and OS rates didn’t differ significantly by primary tumor sites. In this study, the most common primary tumor sites in our population were cervical (45.5%) and rectal (18.2%). We observed that although patients with metastases from cervical cancer achieved possibly faster symptom control and more durable survival compared to those from other origins, the difference was not significant. And more importantly, both cervical and non-cervical cancers presented with satisfactory outcome. The discrepancies between Takeda A *et al*.^19^ and our study could be due to the discrepant tumor differentiation and radioresistant nature of ILNM. Thus, histology might not be a significant predictor of outcome of ILNM, and CyberKnife is applicable to any histological primaries. Randomized prospective trial and laboratory experiment are needed to further assess this.

Typically, leg swelling and pelvic pain are the two most commonly presented symptoms with ILNM. We found that approximately 87% of the patients had symptomatic relief after CyberKnife treatment. When compared to conventional standard radiotherapy of lower daily fraction size as reported by Teh BS *et al*.^20^, the survival was significantly better when symptoms were improved. What is interesting and worth investigating is that since CyberKnife is effective in reducing the already existing symptoms, it might be also applicable to prevent the possible symptoms if administered earlier, as soon as the positive node is found. Besides, CyberKnife was given to 63% of the patients as re-irradiation or boost for recurrence, but no serious toxicity was observed. This is likely due to the highly conformal delivery of radiation to the accurate targets and the large amount of spared nearby normal tissues. However, a prospective trial is needed to confirm these preliminary observations. Settings including fraction, total dose, BED, prior radiotherapy, radiosensitive/radioresistant nature, and DVH of normal tissues are all crucial.

There are some limitations of this study. It is retrospective, and with a limited number of patients or targets. Five males and seventeen females were enrolled, and gender couldn’t match satisfyingly. Otherwise, the treatment schedules were heterogeneous: there was a wide range of doses prescribed for a variety of fractionations. In addition, the follow-up needs to be longer in order to examine the impact of LC on regional or distant disease control and survival.

## Conclusion

CyberKnife SBRT is an effective option with good LC rate, fast symptom relief, and acceptable toxicity for ILNM, especially as re-irradiation or boost for recurrence. Because all the patients are at risk for distant metastasis, local treatment combined with prior systemic treatment will contribute to a better outcome. The treatment plan should be carefully detailed; a total dose more than 30 Gy could improve patients’ survival and for a five-fraction regimen, a prescription dose of at least 35 Gy is recommended to be considered. Histology is not a significant predictor of outcome, thus CyberKnife is applicable to almost any histological primaries. Moreover, further studies in more patients and with longer follow-up is needed to explore the dose-response relationship, the potential toxicity profile, and the chances of long-term survival.

## Materials and Methods

### Patient eligibility

CyberKnife treatment was applied to treat ILNMS in 22 patients with 27 targets in our department from May 2010 to May 2016. All patients were examined by an oncologist before enrollment. The inclusion criteria were defined as follows: ILNM diagnosed by at least an imaging (e.g. CT scan or magnetic resonance imaging (MRI)) ; Karnofsky performance score ≥70; life expectancy of more than 12 weeks; unsuitability for surgery due to old age or poor heart and lung function and received CyberKnife treatment. Informed consent was obtained from all patients. The study was conducted according to the principles expressed in the Declaration of Helsinki and prior approval was obtained from the Medical Ethics Committee of Tianjin Medical University Cancer Institute and Hospital.

### Treatment schedule

Patients were immobilized in the supine position with arms over the head using a thermoplastic body mask and a styrofoam block provided abdominal compression to minimize internal organ motion (spontaneous or breath-induced). CT scan was performed with a slice thickness of 1.5 mm, and the images had to have enough margins (about 15 cm) above and below the tumor according to pretreatment planning CT, PET-CT, and MRI. The gross tumor volume (GTV) was delineated in the treatment planning system and expanded by 3 mm to form the planning target volume (PTV). Three patients were treated with gold fiducial tracking, one with synchrony respiratory motion tracking, and the rest eighteen with X-sight spine tracking. Preventive radiation was not administered to adjacent lymph nodes. The dose-volume constraints for organs at risk were: bladder, V_5mL_ < 37 Gy and D_max_ < 38 Gy; rectum, V_5%_ < 38 Gy and V_2mL_<38 Gy; femoral head, V_30_ < 10 mL.

### Response evaluation and follow-up

Patients were re-evaluated every 6 weeks after CyberKnife treatments. Imaging techniques such as contrast-enhanced CT scans, PET-CT scans or MRI, adverse events, and compliance were monitored. Acute and late toxicity was scored according to the Common Terminology Criteria for Adverse Events, version 4.0^21^. LC was defined as complete response and partial response, and local tumor response was defined using the Response Evaluation Criteria in Solid Tumors (RECIST), version 1.1^22^. OS was defined as the time between the date of the receipt of CyberKnife treatment and the date of death or date of last follow-up for censored patients.

### Statistical analysis

LC and OS curves were estimated using a Kaplan-Meier analysis and compared using the stratified log-rank test. Univariate and multivariate analyses were performed using a Cox regression model. The data were processed using SPSS 18.0 statistical software, and the level of significance was defined as a two-tailed P < 0.05.

## Additional Information

**How to cite this article**: Wang, Z. *et al*. Stereotactic body radiation therapy induces fast tumor control and symptom relief in patients with iliac lymph node metastasis. *Sci. Rep.* **6**, 37987; doi: 10.1038/srep37987 (2016).

**Publisher's note:** Springer Nature remains neutral with regard to jurisdictional claims in published maps and institutional affiliations.

## Figures and Tables

**Table 1:** Patient characteristics.

Characteristics	Values
Age (years)	53 (range 37–87)
Gender (male/female)	5/17 (22.7%/77.3%)
Karnofsky performance score ≧ 70	22 (100%)
Primary tumor	
Cervical cancer	10 (45.5%)
Rectal cancer	4 (18.2%)
Ovarian cancer	2 (9.1%)
Ureter carcinoma	2 (9.1%)
Hodgkin lymphoma	2 (9.1%)
Duodenal cancer	1 (4.5%)
Renal pelvic carcinoma	1 (4.5%)
Symptoms	
Presented	15 (68.2%)
None	7 (31.8%)
Contemporary with other site metastases	
Yes	9 (40.9%)
No	13 (59.1%)
Prior external beam irradiation	
Yes	14 (63.6%)
None	8 (36.4%)
Prior chemotherapy	
Yes	19 (86.4%)
None	3 (13.6%)
Lesions per patient (N)	
=1	19 (86.4%)
>1	3 (13.6%)

**Table 2:** Treatment parameters.

	All lesions	Lesions with local control	Lesions with symptom improved
PTV (ml)	18.87 (0.88–125.66)	16.67 (0.88–125.66)	18.87 (0.88–125.66)
Total prescribed dose (Gy)	39 (21–51)	40.5 (21–51)	39 (21–51)
Number of fractions	5 (3–8)	5 (3–8)	5 (3–8)
Dose per fraction (Gy)	8 (5–13)	7.75 (5–13)	7.5 (5–13)
BED_10_ (Gy)	72.6 (35.70–100)	74.1 (35.70–94.35)	72.6 (35.70–100)
Prescription isodose line (%)	72 (64–82)	71.5 (64–80)	72 (64–82)

**Table 3:** Toxicities.

Toxicity	Total N (%)
Nausea	
Grade 1-2	4 (18.2%)
Grade 3 or more	0 (0%)
Vomiting	
Grade 1-2	3 (13.6%)
Grade 3 or more	0 (0%)
Fatigue	
Grade 1-2	5 (22.7%)
Grade 3 or more	0 (0%)
Abdominal/pelvis pain	
Grade 1-2	2 (9.1%)
Grade 3 or more	0 (0%)
Diarrhea	
Grade 1-2	1 (4.5%)
Grade 3 or more	0 (0%)
Leucopenia	
Grade 1-2	1 (4.5%)
Grade 3 or more	0 (0%)
Thrombocytopenia	
Grade 1-2	0 (0%)
Grade 3 or more	0 (0%)
Intestinal stenosis	1 (4.5%)
